# LONELINESS PREDICTS REDUCTIONS IN SLEEP QUALITY IN OLDER AMERICANS

**DOI:** 10.1093/geroni/igz038.1946

**Published:** 2019-11-08

**Authors:** Sarah Griffin, Baylor Williams, Scott Ravyts, Joseph Dzierzewski, Bruce Rybarczyk

**Affiliations:** Virginia Commonwealth University, Richmond, Virginia, United States

## Abstract

Research documenting loneliness as a factor predicting health decline accumulates, yet the mechanisms underlying this relationship remain obscure. A potential mechanism is sleep disturbance, which is associated with loneliness. However, it remains unclear whether loneliness is a risk factor for subsequent sleep disturbance. The present study aimed to examine loneliness (measured via the Hughes Loneliness Scale) as a risk factor for sleep disturbance in a nationally representative sample of older (>65) Americans. Weighted linear regressions (accounting for complex sampling) were conducted on data from the Health and Retirement Study (n=3,042; 2006 & 2012 waves). Higher levels of loneliness in 2006 predicted sleep disturbance in 2014 when controlling for baseline sleep (B=.08, 95% confidence interval [CI]=[.04, .13]). This association remained after controlling for age, gender, race, ethnicity, education, net worth, and depressive symptoms (B=.07, 95% CI=[.04, .11]). These results identify loneliness as a risk factor for sleep disturbance over an eight-year span in older Americans. Further research is necessary to tease apart this relationship: specifically, to assess reciprocal effects over multiple timepoints, investigate the role of depression in loneliness and sleep disturbance, and employ experimental methods to address causality.

